# Goldenhar Syndrome - ophthalmologist's perspective

**Published:** 2018

**Authors:** Speranţa Schmitzer, Miruna Burcel, Dana Dăscălescu, Ioana Claudia Popteanu

**Affiliations:** * Emergency Eye Hospital, Bucharest, Romania

**Keywords:** Goldenhar, coloboma, epibulbar choristoma, subconjunctival dermoid

## Abstract

Goldenhar syndrome (oculo-auriculo-vertebral dysplasia, OAVS) is a rare, congenital disease arising from the abnormal development of the first and second branchial arches.

The incidence is between 1:3500 and 1:5600, with a male: female ratio of 3:2. The etiopathogenesis is multifactorial and dependent on genetic and environmental factors but there are still many unknown aspects.

The classic features of Goldenhar syndrome include ocular anomalies - epibulbar dermoids, microphthalmia and coloboma, ENT features such as preauricular tragi, hearing loss, low implantation of the auricular pavilion, micrognathia, and vertebral anomalies such as scoliosis or hemivertebrae. The abnormalities are unilateral in 85% of the cases. Ocular features, especially bilateral dermoids are seen in 60% of the cases.

The treatment varies with age and systemic associations, from mainly cosmetic, in uncomplicated cases, to complex reconstructive surgeries in severe cases. While the oculoplastic surgeon manages the oculo-palpebral defects, severe forms require a multidisciplinary approach.

Treatment should be individualized, adapted to age, as well as to the extent and severity of the disease.

The paper is based on the editorial team cases and experience.

## Introduction

Described for the first time in 1952 by Dr. Maurice Goldenhar, Goldenhar syndrome is a type of Craniofacial microsomia (CFM) [**1**].

Goldenhar syndrome (oculo-auriculo-vertebral dysplasia, OAVS) is a rare, congenital disease arising from the abnormal development of the first and second branchial arches [**1**].

Goldenhar syndrome is characterized by a **classical triad**: 1) mandibular hypoplasia resulting in facial asymmetry, 2) ocular and auricular malformations and 3) vertebral anomalies [**1**,**7**].

The **incidence** of this syndrome has been reported to be 1:3500 - 1:5600 with a male to female ratio of 3:2 and it is mostly unilateral in occurrence in 85% of the cases, with the right side being more frequently affected than the left with a ratio of 3:2 [**1**,**4**]. 

The **etiopathogenesis** of this condition is multifactorial, not yet fully established and involves genetic and environmental factors that cause disturbances in neural crest division, abnormal development of the first and second branchial arches during embryogenesis as well as occlusion of placental vessels [**4**,**9**,**11**].

**Genetic factors:** in sporadic cases, 5p deletions, 14q23.1 duplications, or abnormalities of chromosomes 18 and 22 were observed. Families with autosomal dominant inheritance (1-2%) have shown segregation of chromosome 14q23.1 duplication inclusive of the OTX2 gene [**8**,**9**,**11**].

**External factors:** the use of vasoactive drugs during pregnancy (pseudoephedrine, aspirin, ibuprofen) or maternal ingestion of Accutane during the first trimester of pregnancy, maternal second trimester bleeding, gestational diabetes mellitus, multiple gestation and maternal use of assisted reproductive technology are the most common external factors involved in the occurrence of Goldenhar syndrome [**6**,**11**].

Although the **diagnosis** is based primarily on the clinical criteria - phenotypic appearance, radiographic investigations (Rx, CT, and MRI), genetic tests help support the clinical diagnosis. Prenatal diagnosis is possible with considerable accuracy with ultrasound, which may detect obvious defects [**6**].

**Differential diagnosis** is performed with other entities included in the CFM group - CHARGE Syndrome, Parry Romberg Syndrome, or Treacher Collins Syndrome [**4**,**6**].

**Fig. 1 (a,b):**
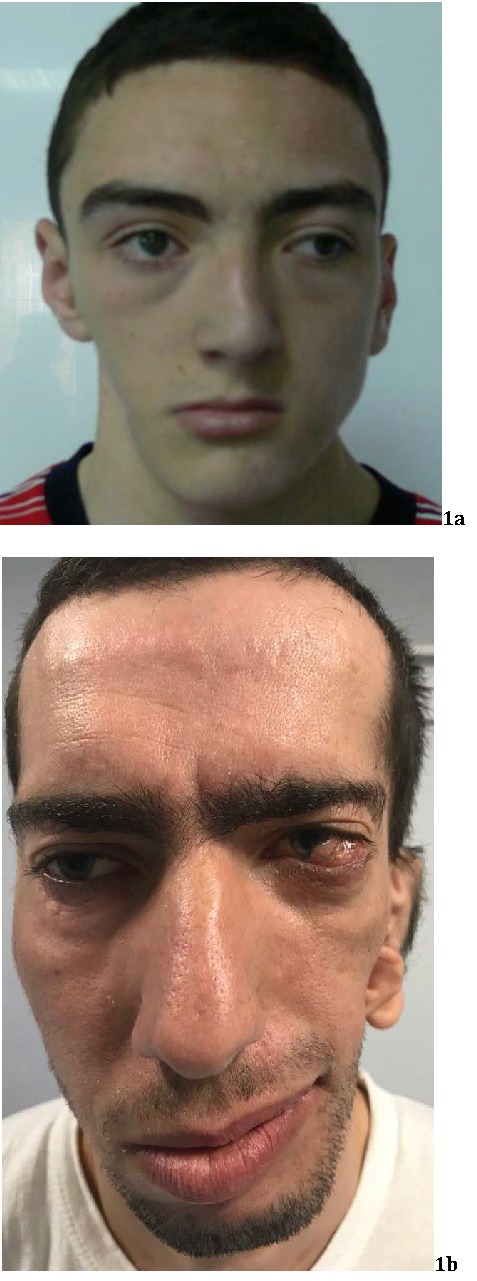
The characteristic combination of external ear, ocular anomalies and ipsilateral facial underdevelopment is the hallmark of Goldenhar syndrome

**Clinically**, Goldenhar syndrome is characterized by: **facial asymmetry**, hemifacial microsomia (80-99%) (**Fig. 1**); **ENT anomalies:** accessory tragi (80-99%), hypoplasia of the maxilla, malar flattening (80-99%), cheilopalatoschisis, low-set, posteriorly rotated ears, laryngomalacia; **vertebral anomalies:** hemivertebrae, scoliosis; **cardiac anomalies** - Tetralogy of Fallot, ventricular septal defects, transposition of the great vessels; **CNS malformations** - microcephaly, encephalocele, hydrocephaly, hypoplasia of the corpus callosum, Arnold-Chiari malformation; and last but not least **ocular anomalies** [**5**,**6**,**11**].

**Ocular features**

**Fig. 2:**
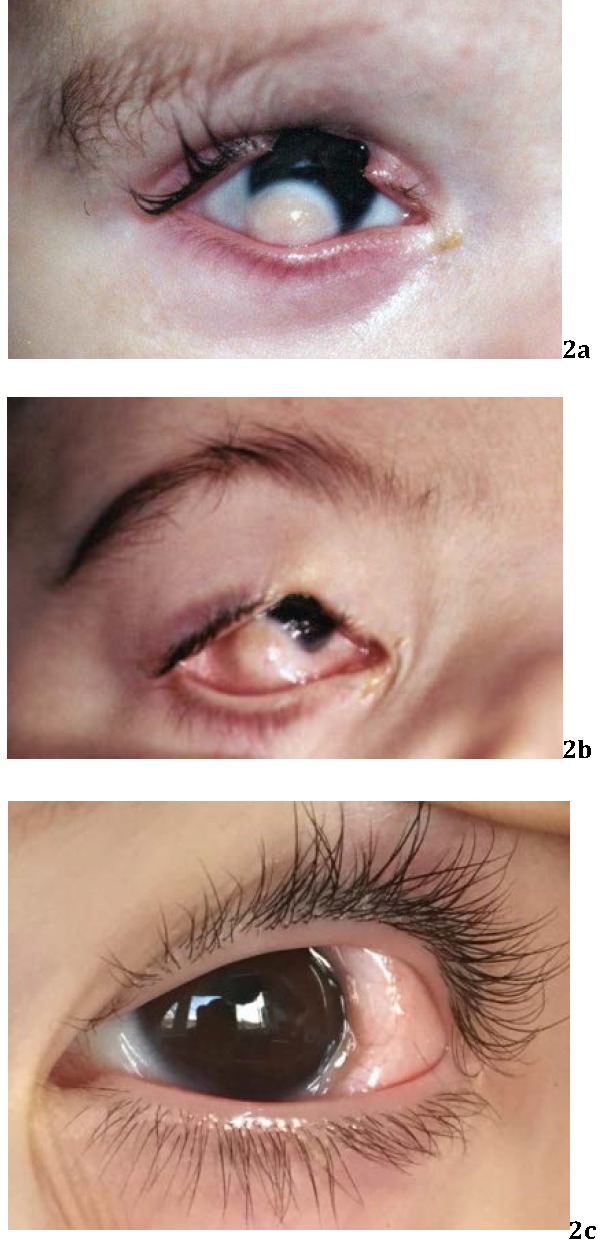
Ocular features in Goldenhar Syndrome – upper eyelid coloboma, epibulbar choristoma (a, b), subconjunctival dermoid (c)

They are most commonly represented by upper eyelid colobomas (**Fig. 2 a,b**) associated with iris/ chorioretinal coloboma, epibulbar choristoma, subconjunctival dermoid (**Fig. 2 c**) and less frequently - microphthalmia/ anophthalmia, strabismus, cataract or inequality of palpebral fissures [**6**,**11**].

**Upper eyelid coloboma - CLINICAL-SURGICAL EMERGENCY**

**Fig. 3:**
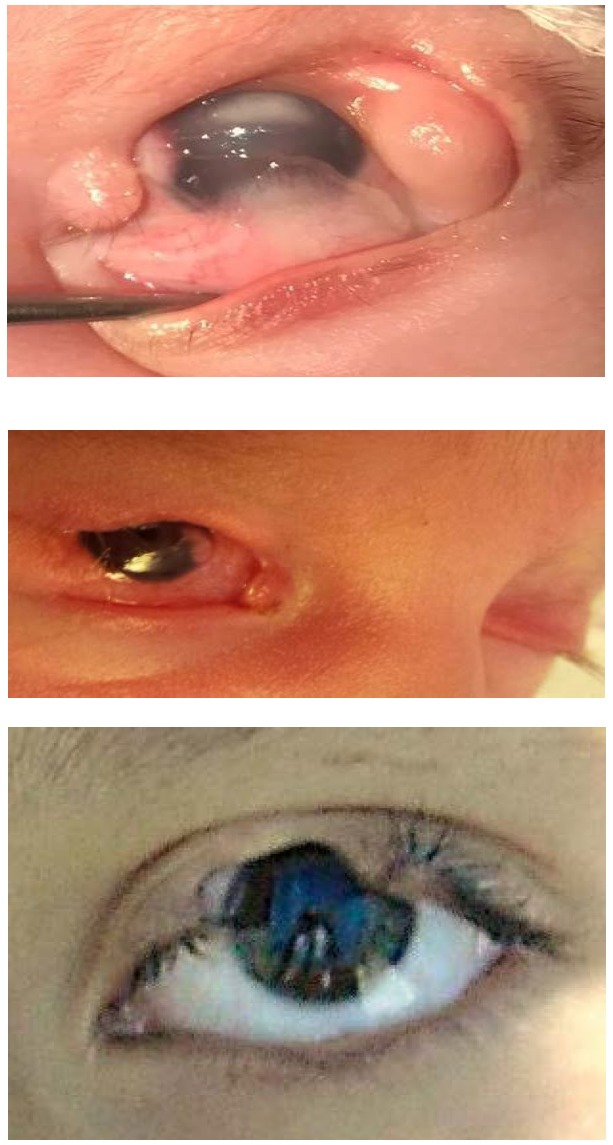
Upper eyelid coloboma

The upper eyelid coloboma (**Fig. 3**) is characterized by differently sized lack of substance in the entire thickness of the eyelid. The most frequent location is the junction of the middle and inner thirds [**2**].

**Fig. 4:**
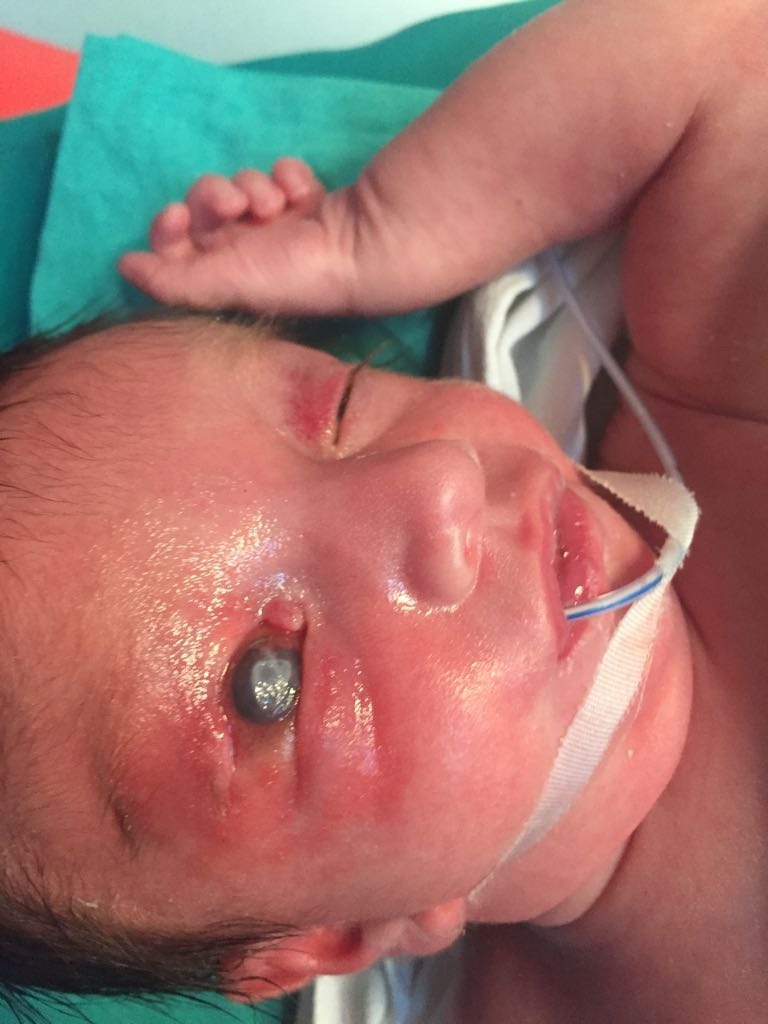
RE upper eyelid coloboma complicated with corneal ulceration

The upper eyelid coloboma is a surgical emergency since the first days of life because it can lead to serious ocular complications - corneal ulceration (**Fig. 4**), corneal perforation, corneal leukoma, and amblyopia [**2**].

**Management of congenital eyelid coloboma**

The first and most important step in the therapeutic management of an eyelid coloboma is the corneal protection - therapeutic contact lens, eye lubricating drops/gels/ointments. Avoid eye bandaging!

The palpebral reconstruction is essential both from a functional and an aesthetic point of view. The time of surgery depends on the size of the coloboma, its location, the risk of corneal exposure, the general state of health or associated diseases of the child [**2**]. 

If the defect is small and there is no corneal exposure, surgery may be delayed. Otherwise (if the defect exceeds 1/3 of the length of the eyelid and there is corneal exposure), we must intervene as soon as possible to avoid permanent corneal damage.

The surgical approach and technique depend on the size of the coloboma.

In choosing the surgical technique we must take into account the “quarter” rule - a lack of substance equal to one quarter of the length of the eyelid does not alter the static, dynamic and aesthetics of the eyelid.

**Fig. 5:**
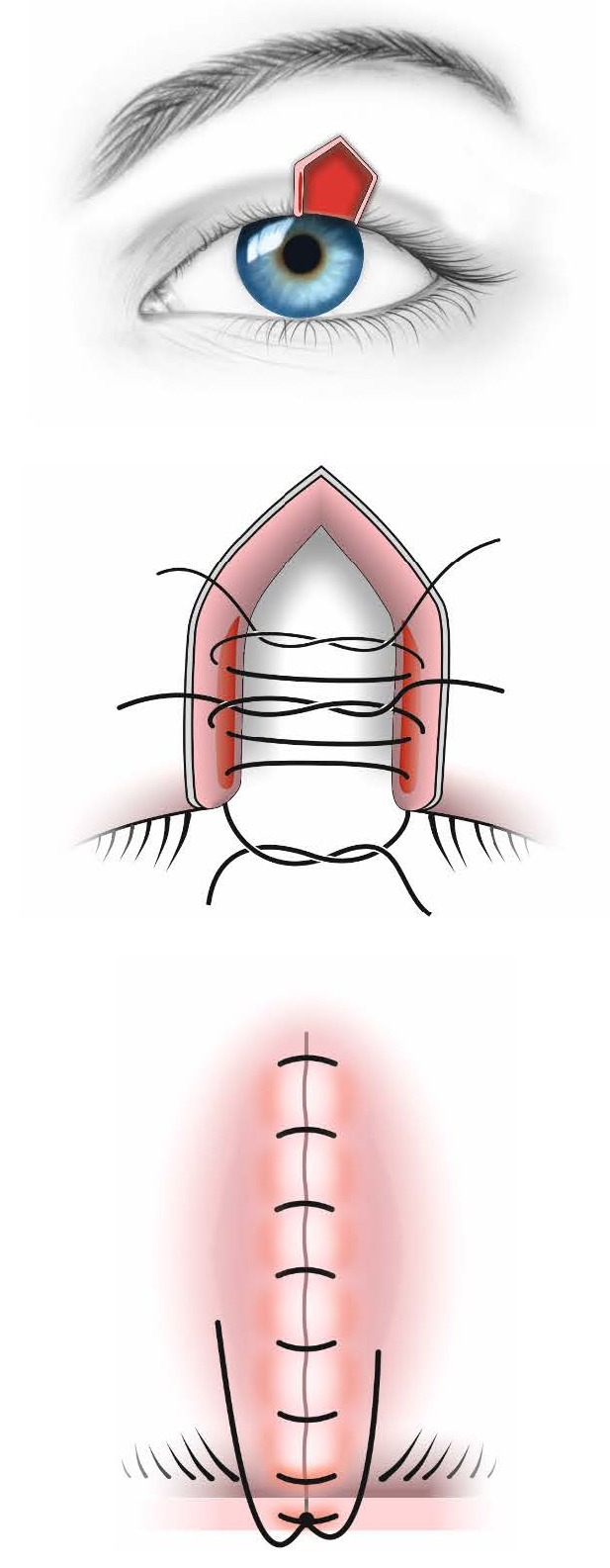
Small defect - up to 25% - direct tissue apposition

**Fig. 6:**
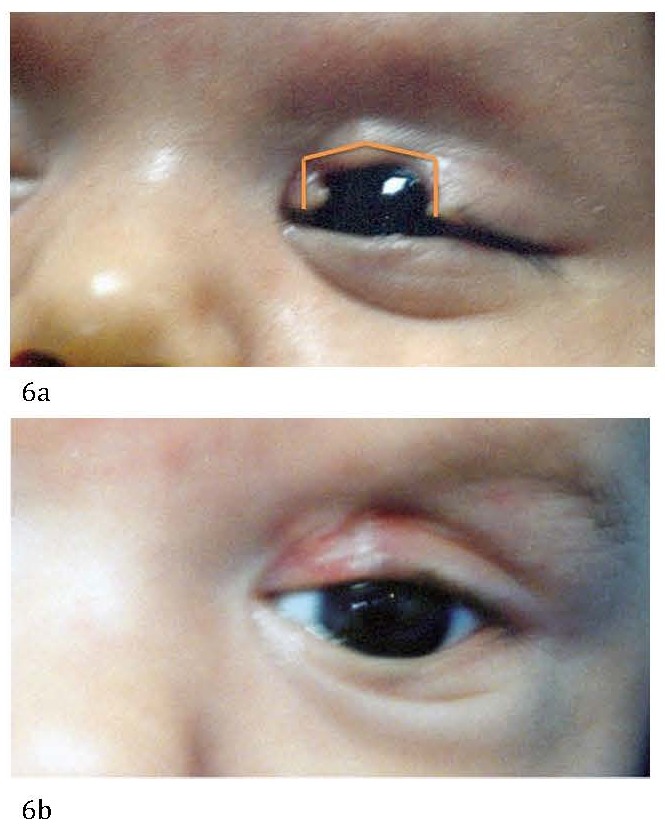
Upper eyelid coloboma before (a) and after (b) direct tissue apposition.

For small defects (**Fig. 5**,**6**), that do require suturing (up to 25%), direct tissue apposition (after the excision of the coloboma margins) may suffice [**2**].

**Fig. 7:**
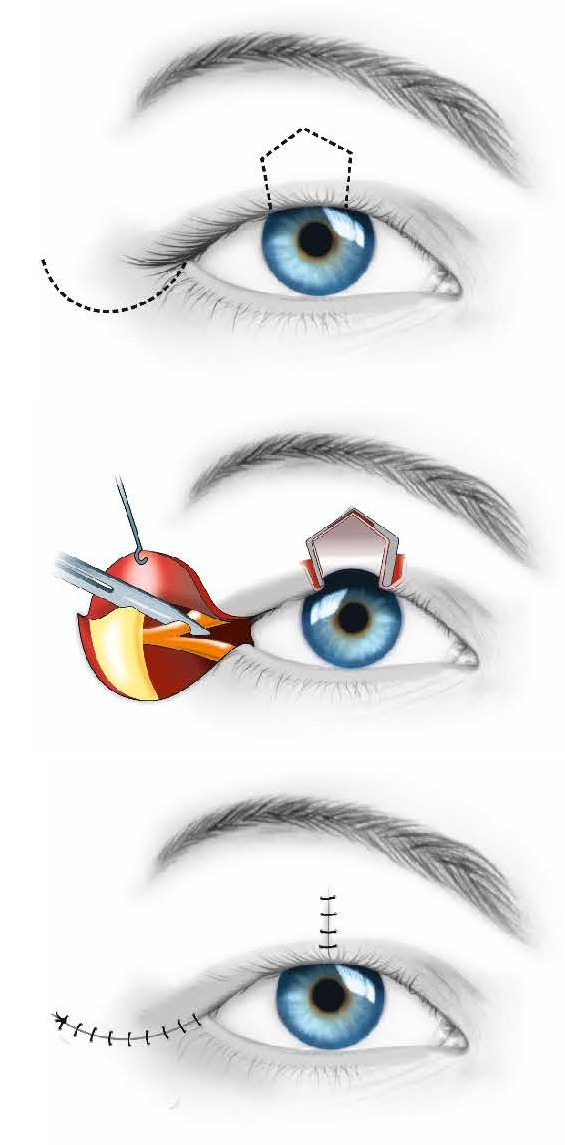
Coloboma between 25-35% of the length of the eyelid - Tenzel semicircular flap

**Fig. 8:**
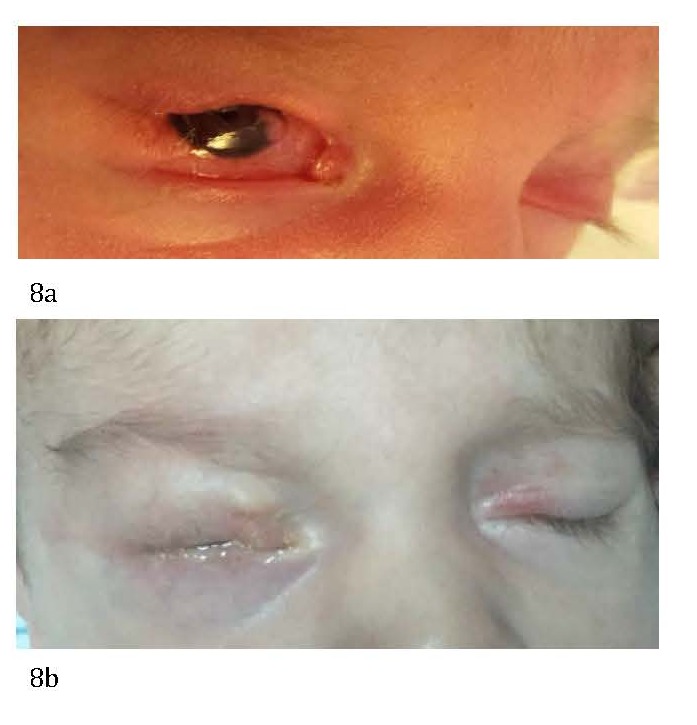
Large upper eyelid coloboma before (a) and after (b) a Tenzel semicircular flap was performed

If the size of the coloboma ranges between 25 and 35% of the length of the eyelid (**Fig. 7**), a Tenzel semicircular flap (**Fig. 8**) should be used [**2**].

**Fig. 9:**
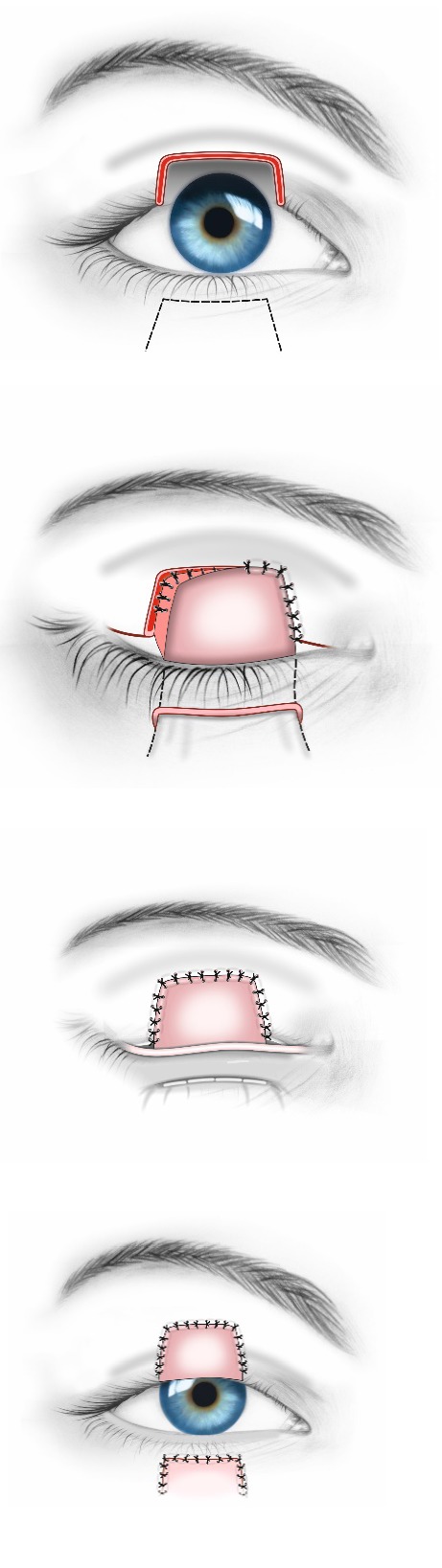
Cutler Beard technique

**Fig. 10:**
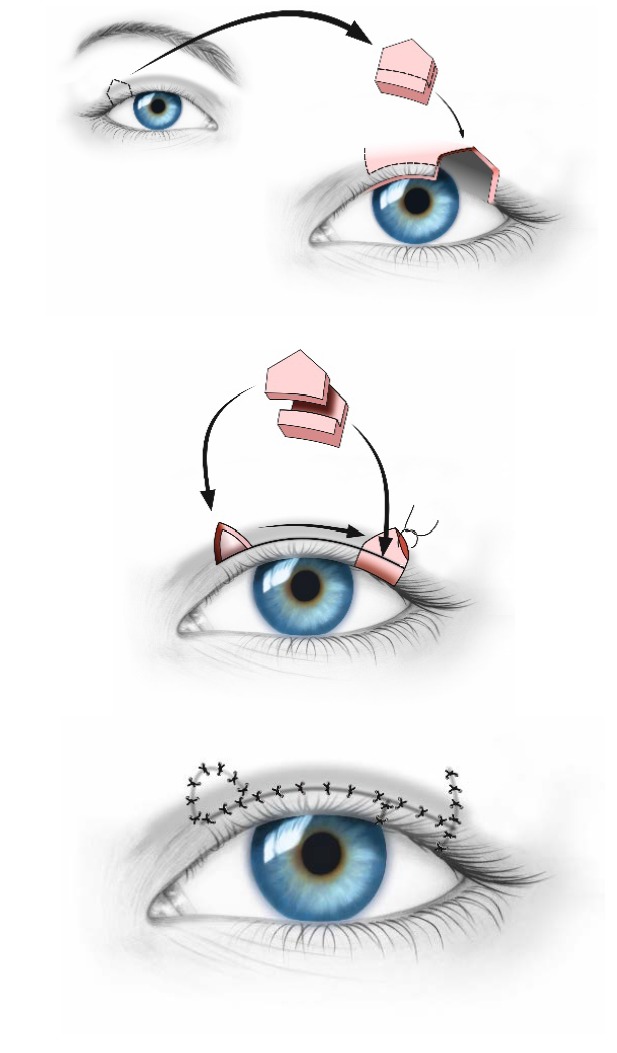
Tarsomarginal graft

With large colobomas (> 35% of the eyelid length), functional and aesthetic results are difficult to obtain. Different techniques are used: Cutler Beard technique (**Fig. 9**), Mustard rotational flap, tarsomarginal grafts (**Fig. 10**), modified Hughes procedure. The main disadvantage of these techniques is represented by temporary palpebral occlusion with the risk of amblyopia, which is why careful monitoring of the patients is required [**2**,**10**].

Coloboma > 35%:

**Epibulbar choristoma**

**Fig. 11:**
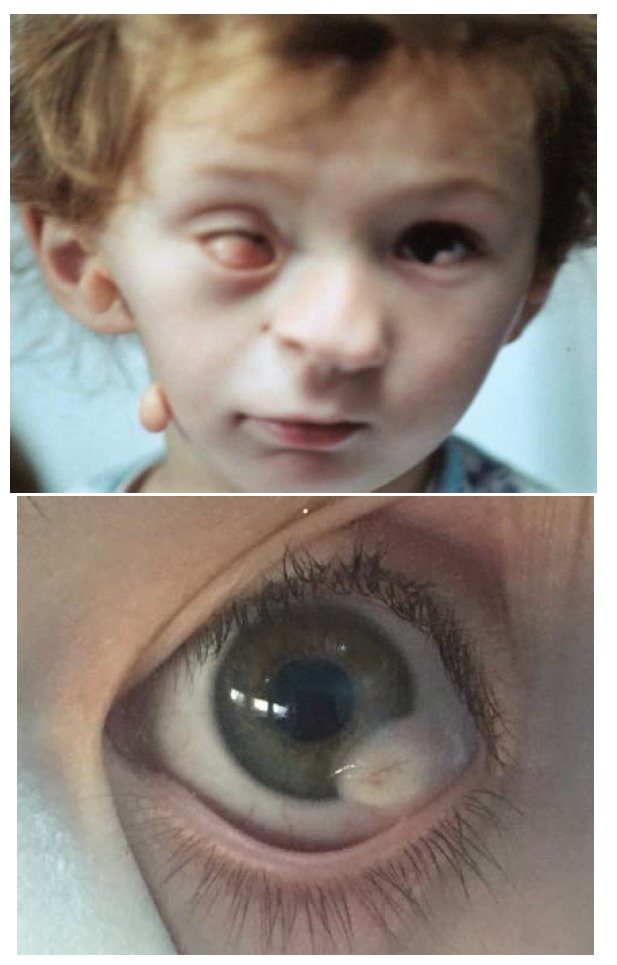
a- Bilateral epibulbar choriostomas; b – epibulbar choriostoma with hair follicles on the surface

The hallmark of ocular features in Goldenhar syndrome is the presence of an eccentric or limbal epibulbar dermoid - soft, localized, elevated, opaque, yellow-white mass at limbus, with full thickness corneal involvement, sometimes with hair follicles on the surface (**Fig. 11**). The most common location is inferotemporal, but nasal limbal choristomas have been reported [**3**].

Current standard medical treatment for grade I pediatric limbal dermoids (with superficial corneal involvment) is initially conservative. In stages II (ie, affecting the full thickness of the cornea with/without endothelial involvement) and III (ie, involvement of entire cornea and anterior chamber), a combination of excision, lamellar keratoplasty, and amniotic membrane and limbal stem cell transplantation are advocated [**3**]. 

The purpose of treatment is to prevent amblyopia (caused by induced astigmatism or obstruction of the visual axis) or aesthetic considerations.

**Fig. 12:**
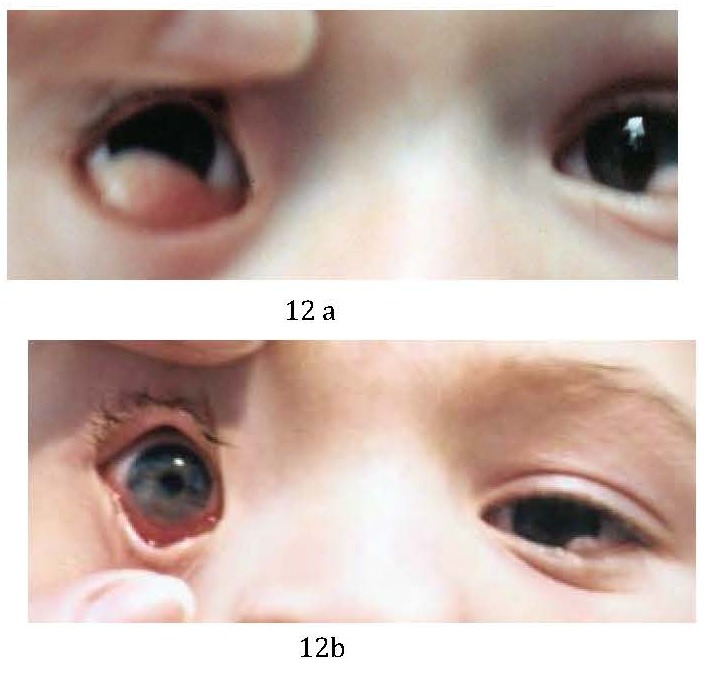
Epibulbar choriostoma berfore (a) and after (b) surgical excision.

**Lipodermoids (dermolipomas)**

**Fig. 13:**
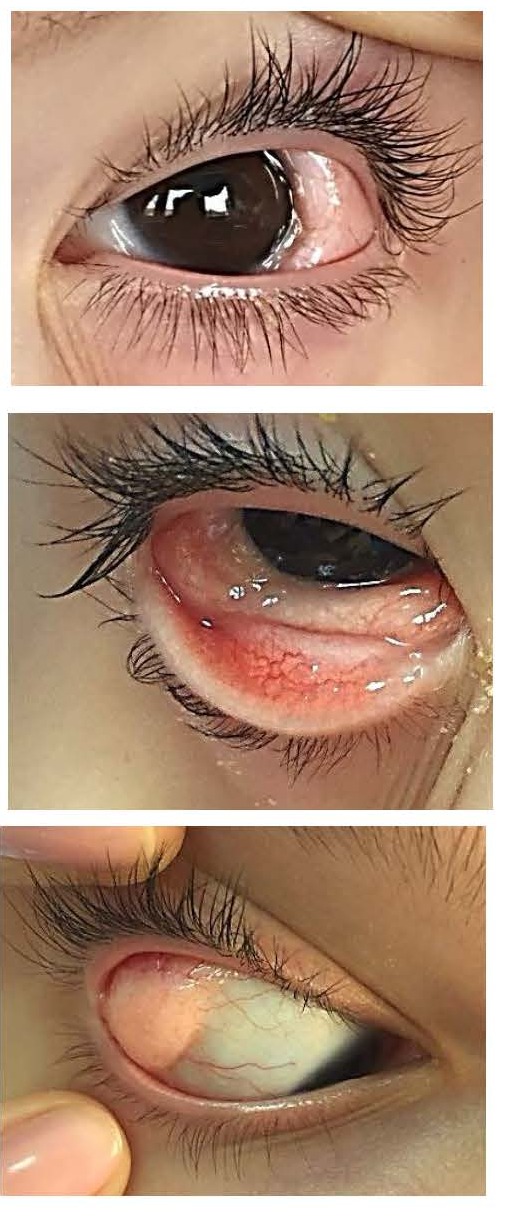
Dermolipomas

**Fig. 14:**
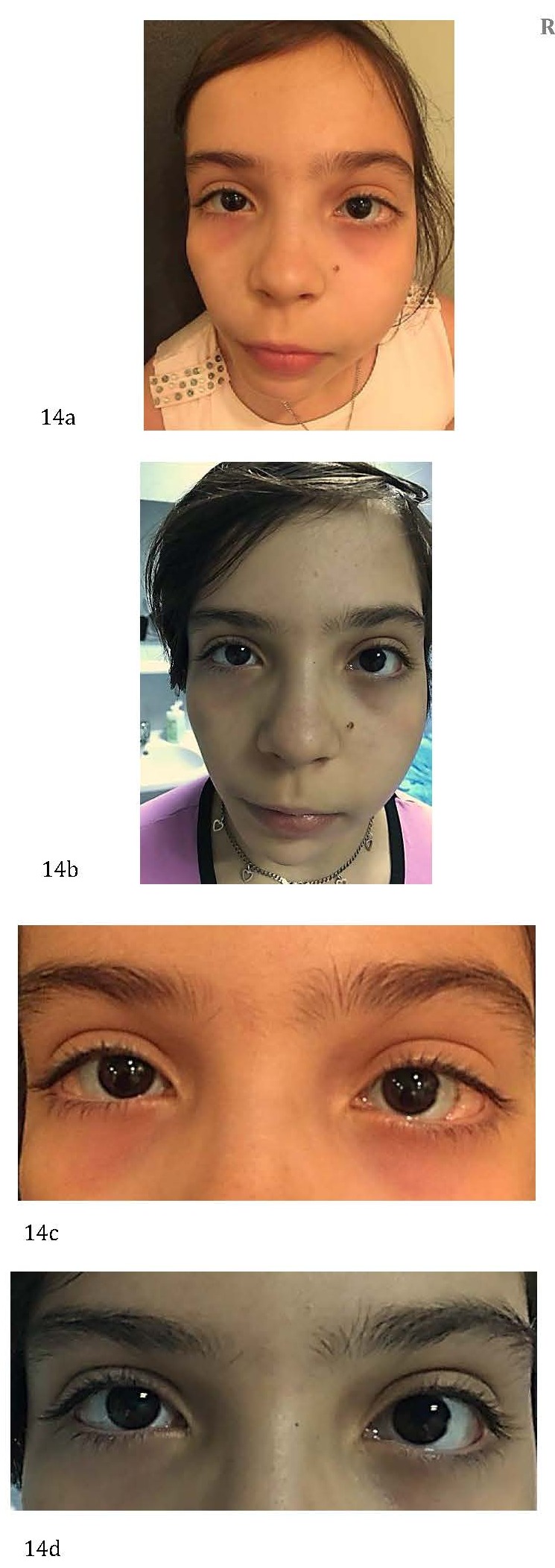
Hemifacial microsomia and bilateral lipodermoids – before (a,c) and after (b,d) the surgical excision

Strikingly more yellowish than epibulbar dermoids (because of a substantial deep layer of mature fat cells), subconjunctival lipodermoids (**Fig. 13**,**14**) are not only limited to the superotemporal quadrant but may also occur inferiorly [**9**].

Surgical treatment (simple excision and conjunctivoplasty – completed with great care to prevent underlying muscle damage or shortening of conjunctival fornices) is mainly performed for cosmetic reasons, and rarely for functional reasons (globe movement restrictions, visual axis obstruction) [**9**].

## Conclusions

Goldenhar syndrome is a relatively rare condition, easy to diagnose based on the clinical appearance without excluding further investigations. Surgical treatment is mandatory, complex, individualized, and sometimes multidisciplinary, with very good cosmetic and functional results.

The treatment of eyelid coloboma is a CLINICAL-SURGICAL EMERGENCY (from the first day of life) in order to preserve the cornea and visual function, requiring a well-coordinated teamwork between neonatologists and ophthalmologists.

Epibulbar choristomas should be excised in the first years of life, followed by appropriate refractive correction to avoid amblyopia.

Lipodermoids are considered surgical emergencies only if they cause strabismus.

Although it is not possible to specify the optimal moment of surgery, its postponement does not bring benefits aesthetically or functionally. Fast intervention in the first years of life provides the patient a faster recovery and a better quality of life.

Finally, yet very important, is paying increased attention to postoperative care in order to avoid the occurrence of complications (wound dehiscence) and to obtain the best aesthetic and functional result.
